# Dual‐tracer‐based isotope turnover rates in a highly invasive mysid *Limnomysis benedeni* from Lake Constance

**DOI:** 10.1002/ece3.2928

**Published:** 2017-04-28

**Authors:** Elizabeth Yohannes, Karl‐Otto Rothhaupt

**Affiliations:** ^1^ Limnological InstituteUniversity of KonstanzKonstanzGermany

**Keywords:** Na^15^NO_3_, NaH^13^CO_3_, stable isotope, δ^13^C, δ^15^N

## Abstract

Understanding the ecological patterns of invasive species and their habitats require an understanding of the species’ foraging ecology. Stable carbon (δ^13^C) and nitrogen (δ^15^N) isotope values provide useful information into the study of animal ecology and evolution, since the isotope ratios of consumers reflect consumer's dietary patterns. Nevertheless, the lack of species‐ and element‐specific laboratory‐derived turnover rates could limit their application. Using a laboratory‐based dual stable isotope tracer approach (Na^15^ NO_3_ and NaH^13^ CO_3_), we evaluated the δ^15^N and δ^13^C isotope turnover rates in full‐grown adult invasive *Limnomysis benedeni* from Lake Constance. We provide δ^15^N and δ^13^C turnover rates based on nonlinear least‐squares regression and posterior linear regression models. Model precisions and fit were evaluated using Akaike's information criterion. Within a couple of days, the δ^15^N and δ^13^C of mysids began to change. Nevertheless, after about 14 days, *L. benedeni* did not reach equilibrium with their new isotope values. Since the experiment was conducted on adult subjects, it is evident that turnover was mainly influenced by metabolism (in contrast to growth). Unlike traditional dietary shifts, our laboratory‐based dual stable isotope tracer approach does not shift the experimental organisms into a new diet and avoids dietary effects on isotope values. Results confirm the application of isotopic tracers to label mysid subpopulations and could be used to reflect assimilation and turnover from the labeled dietary sources. Field‐based stable isotope studies often use isotopic mixing models commonly assuming diet‐tissue steady state. Unfortunately, in cases where the isotopic composition of the animal is not in equilibrium with its diet, this can lead to highly misleading conclusions. Thus, our laboratory‐based isotopic incorporation rates assist interpretation of the isotopic values from the field and provide a foundation for future research into using isotopic tracers to investigate invasion ecology.

## 1 Introduction

Stable isotope analysis (SIA) has been a routine tool in animal ecology and evolution, especially for estimation of trophic positions: A key parameter to understand food web structure in natural ecosystems (Gannes, O'Brien, & Martínez del Rio, 1997; Martínez del Rio, Wolf, Carleton, & Gannes, 2009). SIA is used in nutrition studies and dietary reconstruction such as those that investigate temporal change in diet, to evaluate contribution of dietary nitrogen or carbon sources, tissue‐specific protein turnover rate, and spatio‐temporal relationships between prey and consumers (e.g., Martínez del Rio & Anderson‐Sprecher, 2008; Tieszen, Boutton, Tesdahl, & Slade, 1983). One of the advantages of SIA is that, contrary to traditional methods, it can reconstruct assimilated dietary sources over various time scales (e.g., Tieszen et al., 1983). This reflects temporal isotopic dynamics: Notably the principle that an animal tissue does not immediately reflect its dietary isotopic composition, but integrates over some period of time scale (Martínez del Rio & Anderson‐Sprecher, 2008).

To ensure correct SIA application in invasive biological or ecological studies, one must include appropriate estimates of isotopic turnover rates and changes in tissues following shifts in stable isotope values. Thus, knowledge of elemental turnover rates (*t*_1/2_) is decisive, as precise turnover rate values allow reliable estimates of diet contribution and a reasonable approximation of prey‐consumer relationship in different diet or feeding regimes (Boecklen, Yarnes, Cook, & James, 2011). Lack of appropriate turnover rate values might result in over‐ or underestimation and thus erroneous interpretation of stable isotope values. As such, most studies lack such values and apply approximated estimates obtained from published values or reviews (e.g., Caut, Angulo, & Courchamp, 2009).

Available data indicate that these values might vary between tissue types, species or between individuals (Kaufman, Gradinger, Bluhm, & O'Brien, 2008; Marín Leal et al., 2008; Suring & Wing, 2009) and between dietary quality and quantity (Adams & Sterner, 2000; Caut et al., 2009). Most of the studies on isotopic turnover rates have been conducted in vertebrates (fish, birds, and mammals, reviewed in Boecklen et al., 2011) and even so, there is a call for more laboratory experiments (Gannes et al., 1997; Martínez del Rio et al., 2009). In short, there is insufficient knowledge on how stable isotope turnover rates vary between individuals of a given species held under different or similar environmental and/or individual conditions.

In studies that deal with ecology and evolution of invasive species, SIA is often used to understand competitive interactions between invaders and functionally similar native species (e.g., Jackson et al., 2016). Despite its potential to evaluate diet efficiency in native and non‐native species, SIA has rarely been used to analyze diets in invasive species such as the crustacean *Limnomysis benedeni* (Macostraca: Peracardia: Mysida), one of the most widespread biological invaders in freshwater systems globally (Audzijonyte, Wittmann, Ovcarenko, & Väinölä, 2009; Băcescu, 1954; Borza, 2014; Rothhaupt, Hanselmann, & Yohannes, 2014). Fully grown adult *L. benedeni* tissues are metabolically active, and there is therefore the potential for turnover of isotope values to assist in understanding diet types and feeding scenarios of adult invasive mysids. Changes in the isotope values with dietary treatment as a function of time are necessary for accurate estimation of mixing models and models that apply nutritional studies of invader mysids and functionally similar native and non‐native species.

Therefore, the main objective of our study was to determine of stable nitrogen (δ^15^N) and carbon (δ^13^C) isotopes in adult invasive mysids. Using a laboratory‐based dual stable isotope tracer approach (Na^15^NO_3_ and NaH^13^CO_3_), we tested how the stable isotope values adult individual *L. benedeni* shift over a 2‐week period when exposed to a new isotopic environment. In addition to the effect this invader species could exert on benthos, biofilm communities, and small pelagic bioseston and abioseston (such as plankton, nekton, and detritus), it represents a suitable food resource for higher trophic consumers, such as fish (Hanselmann, Gergs, & Rothhaupt, 2011a, 2011b).

The above considerations indicate that invasive *L. benedeni* may affect the food web through both “bottom‐up” and top‐down” processes. Understanding *L. benedeni* invasion ecology depends on accurate reconstructions of *L. benedeni* diets. Direct study of their diet in wild and laboratory condition has been challenging, because *L. benedeni* are difficult to observe in the wild and they digest different types of prey at different rates; and accurate identification of stomach contents could be difficult (Fink & Harrod, 2013; Gergs, Hanselmann, Eisele, & Rothhaupt, 2008; Hanselmann, Hodapp, & Rothhaupt, 2013; Rothhaupt et al., 2014).

## 2 Methods

### 2.1 Algal cultivation

Two cultures of Eustigmatophytes, *Nannochloropsis limnetica* (SAG 18.99), were cultivated semi‐continuously at a dilution rate of 0.2 g/day in aerated 5‐L vessels containing Woods Hole (WH) medium (20°C, illumination at 120 μmol quanta m^−2^ s^−1^; Guillard, 1975) enriched with vitamins. To be able to grow algae in nitrogen enriched condition, one batch of culture of *N*. *limnetica* was grown in WH medium supplemented with isotopic tracers of 0.1% Na^15^NO_3_ (Sigma‐Aldrich 98%) and 30% NaH^13^CO_3_ (Cambridge Isotope Laboratories, Inc. 99%). A second batch of culture of *N. limnetica* was grown as “normal” culture with no tracer enrichment. Algae were harvested during their late exponential growth phase.

Food suspensions, each with a total carbon concentration of 2 mg C/L, consisting of equivalent amount of *N. limnetica* were prepared by concentrating (centrifugation at 3,000 *g*, 10 min) and resuspending the cells in fresh medium. Food suspension carbon concentrations were estimated by photometric light extinctions (800 nm) and carbon‐extinction equations determined prior to the experiment. The carbon‐light extinction regressions were subsequently confirmed by carbon analysis of the food suspensions using an elemental analyzer (VarioPyrocube, Elementar, Hanau, Germany).

### 2.2 Experimental animal acquisition and care

Adult *L. benedeni* obtained from the littoral zone of Lake Constance using light traps sampling approach at the shoreline of the University of Konstanz, Egg 47°41′46″N; 9°11′31″E). Mean body length (±*SE*) of individual animals was 8.97 (±0.06). Animals were transported to the Limnological Institute of the University of Konstanz. First, to acclimate the organisms to laboratory condition, they were all kept in climate chambers with a diurnal dark‐light cycle of 12 hr: 12 hr at 15°C in aerated 250‐ml glass beakers containing lake water for 2 weeks. Following this acclimation procedure, a 14‐day experiment was initiated in the same condition using prefiltered lake water (0.2 μm pore‐size membrane filter), with each beaker containing a maximum of five individuals. Animals were kept in two groups: While a “control” group was fed with 5 mg C/L “normal” *N. limnetica*, 5 mg C/L “enriched” *N*. *limnetica* was applied to feed an “enriched” group.

Mysids were transferred into new beakers every second day to avoid accumulation of pellets and biofilm formation. Food suspensions were added with a pipette near the bottom of each beaker to allow the sedimentation of algae and to increase the availability of food particles for the mysids. Before SIA on tissues of experimental animals, mysids were kept overnight in filtered lake water over gauze screens to allow gut clearance. All experiments were conducted from 19th October to the 2nd November (2013), using only sexually matured adults of the winter generation, that are unlikely to invest neither in increasing body size nor in reproduction (Hanselmann et al., 2011a, 2011b). SIA was conducted on replicate samples consisting of single individuals monitored over 2 weeks.

### 2.3 Stable isotope analysis

Dried and powdered samples (ca. 0.76 mg whole body *L. benedeni*, 1 individual per analysis) were loaded into tin capsules and combusted in a vario Micro cube elemental analyzer (Germany). The resulting gases were fed via gas chromatography into the inlet of a micromass (Manchester, UK) isoprime isotope ratio mass spectrometer. Carbon, nitrogen, and sulfur were extracted from precombusted GF/F filters (Whatman, 25 mm diameter; GE Healthcare Life Science, Freiburg, Germany) loaded with approximately 1 mg particulate organic matter of the food suspensions or organic matter obtained from gut clearance.

Measurements are reported in δ‐notation in parts per thousand deviations (‰) relative to international standards for carbon (Pee Dee Belemnite) and nitrogen (atmospheric N_2_, AIR), according to the equation

$$\delta \;\left(‰\right)=1,000\times \left(\frac{R_{\mathrm{sample}}}{R_{\mathrm{standard}}}-1\right).$$

Two sulfanilamides (Iso‐prime internal standards) and two Casein standards were used for every seven unknowns in sequence. Internal laboratory standards indicated measurement errors (*SD*) of ±0.03‰ for δ^13^C and 0.12‰ for δ^15^N.

### 2.4 Stable isotope turnover rates

For individuals that demonstrated a shift in isotope values toward the tracer values and toward equilibrium during the feeding trial, tissue turnover rates were estimated by fitting a nonlinear least‐squares regression model using the following equation:

$$\delta_{t}=\delta_{\mathrm{eq}}+\left(\delta_{o}-\delta_{\mathrm{eq}}\right)e^{-(\lambda )t},$$

where δ_*t*_ isotopic value (‰) at time *t*; δ_o_ initial isotopic value (‰) at equilibrium with the “normal” diet (unlabeled); δ_eq_ isotopic value (‰) after equilibration with the enriched diet (labeled diet); *t* time (days) and λ = turnover rate (days^−1^).

We note that we have taken a limited sample size (maximum of five individuals) per sampling date. This might bias our interpretation. Therefore, *r* ^2^ and akaike's information criterion corrected for small sample sizes (AICc) were determined to evaluate the precision and fit of correction models. The resultant linear model was used to re‐estimate the model values in order to standardize them, and adjusted *t*_1/2_ was determined.

## 3 Results

### 3.1 Stable isotope tracer experiment

Mean δ^15^N and δ^13^C (±*SE*) values of tracer “enriched” diet were 72.43‰ ± 7.02 and 7.06‰ ± 1.71, respectively. Mean δ^15^N and δ^13^C (±*SE*) values of “normal” diet were 7.55‰ ± 0.29 and −26.28‰ ± 0.24, respectively.

#### 3.1.1 Nitrogen and carbon turnover

**Figure 1:**
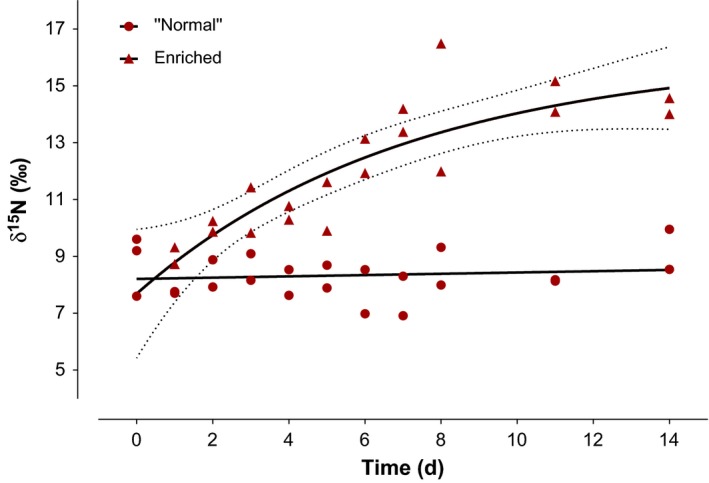
Time‐based δ^15^N values of invasive *Limnomysis benedeni* during a 2‐week experiment. Mysids from Lake Constance, Germany were reared in Na^15^ NO_3_ enriched algal food source in laboratory condition. Whole‐body tissue samples were analyzed

The best fit for δ^15^N values correspond to one‐phase exponential decay (Difference in AICc = 1.578). The δ^15^N values of invasive *L. benedeni* from Lake Constance, Germany experimentally held in δ^15^N enriched cultures of Eustigmatophytes began changing after about 4 days (δ^15^N *T*_0_ = 8.8‰; *T*_4_ = 10.52‰, Figure 1). After that point, the δ^15^N values increased (posterior estimate of slope, *b*, mean = 0.46, 95% CI = [0.30–0.62], *r* ^2^ = .68).

**Figure 2:**
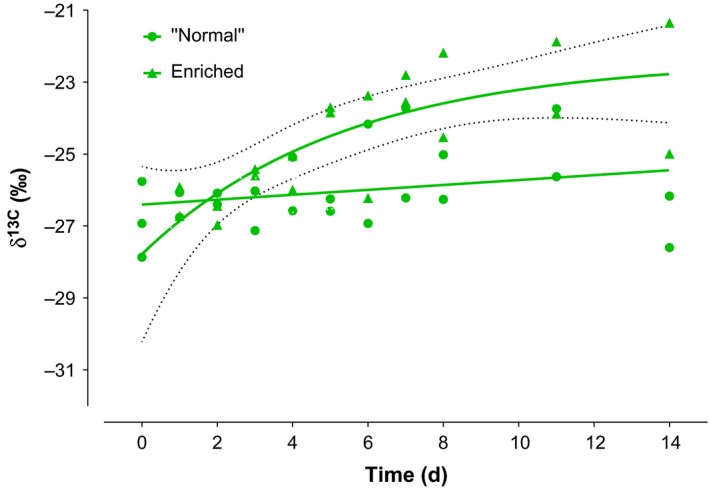
Time‐based δ^13^C values of invasive *Limnomysis benedeni* during a 2‐week experiment. Mysids from Lake Constance, Germany were reared in NaH^13^ CO_3_ enriched algal food source in laboratory condition. Whole‐body tissue samples were analyzed

The preferred model for experimentally enriched δ^13^C values corresponds to one‐phase exponential decay (Difference in AICc = 0.58). The δ^13^C values were evident to significantly change beginning day 5 (δ^13^C *T*_0_ = −26.85‰; T_5_ = −23.78‰, Figure 2). The δ^13^C values increased (posterior estimate of slope, *b*, mean = 0.30, 95% CI = [0.15–0.45], *r* ^2^ = −0.51). The overall slopes between δ^15^N and δ^13^C were identical (*F*_1,36_ = 2.49; *p* = .12).

The preferred model for “normal” diet δ^15^N and δ^13^C values correspond to simple straight line models *f*(*t*) = *m·t + b*, where, *m* is the change in the tissue fractional turnover rate associated with no change in diet, *b* is the initial isotopic value predicted by the linear equation, and *t* is the days since the isotopic switch. As expected, a minor rate of turnover (*m*, mean ± *SE*) for δ^15^N and δ^13^C corresponding to 0.02‰ ± 0.03 (*r* ^2^ = .01) and 0.06‰ ± 0.05 (*r* ^2^ = .07), respectively was calculated (Table 1).

**Table 1:** Parameter estimates from nonlinear least‐squares time‐based δ^15^N and δ^13^C models of *Limnomysis benedeni* whole‐body tissues, including the initial isotope value for that tissue (i.e., day 0 or 1, final value (δ_f_; ‰), isotopic turnover (*m*; day^−1^), proxy to model fit (*r* ^2^)

A. Isotope	Type	δ_i_	δ_f_	*r* ^2^	*m*
δ^15^N	Enriched	7.68	16.10	.74	0.14
δ^13^C	Enriched	−26.79	−22.39	.59	0.14

B. Isotope	Type	Equation	*r* ^2^	*m*
δ^15^N	Normal	*Y* = 0.02*X* + 8.20	.02	0.57
δ^13^C	Normal	*Y* = 0.07*X* − 26.41	.07	0.22

Isotope turnover (*m*) was estimated using one phase exponential decay model (A). Linear regression equation, *r* ^2^ and *p* values for δ^15^N and δ^13^C values of *L. benedeni* whole‐body tissues (B).

## 4 Discussion

Globally, mysids play an important role as the most successful species among aquatic alien (animal) invaders (Hänfling, Edwards, & Gherardi, 2011). Among these, *L. benedeni* is reported as one of the most widespread invaders in Central Europe (e.g., Wittmann & Ariani, 2009). It originates from the area of the Black Sea and the estuaries of the Danube (Audzijonyte et al., 2009; Băcescu, 1954) and has been a threat to several large lakes, including Lake Constance. In fact, the population of *L. benedeni* in Lake Constance is the only population thus far registered from a larger and deep freshwater lake. Our study has a focus on Lake Constance, a prealpine European lake with its shoreline shared among Germany, Switzerland, and Austria.

Traditional approaches to stable isotope experiments that evaluate elemental incorporation rates are often conducted using dietary switch, by altering the “normal” (usual) diet of the study organism into a “new” experiment diet. Such approaches might face disadvantages in that differences in dietary quality between experimental and “normal” (usual) diet might affect stable isotope turnover rates (Caut et al., 2009). Our results confirm equivalent turnover of carbon and nitrogen in mysids indicating comparable rate of utilization of both elements by individual specimen. As such, the tracer experiment did not shift the experimental subjects into a completely new or different diet. Yet, after about 14 days, *L. benedeni* did not reach isotope values expected for equilibrium with their new isotope values.

It is worth remembering that growth is expected to increase turnover in smaller‐sized juveniles and rapidly growing individuals (Fry & Arnold, 1982; Hesslein, Hallard, & Ramlal, 1993). We are aware of the assumption that the stable isotope ratios from tissue subsamples with faster turnover rate (such as internal soft‐tissues or eggs during reproductive season) may reach diet‐tissue steady state more quickly than whole‐organism homogenates (Post, 2002). Nevertheless, individual specimens exhibited shifts in both δ^13^C and δ^15^N. However, relative to the highly enriched tracer values incorporated in their diet, these results likely indicate slower turnover of nitrogen and carbon achieved through algal grazing. Still, the isotopic shift was apparent in these tissues (nitrogen: range = +8.72‰ to +16.48‰; carbon: range = −26.98‰ to −21.36‰) indicating that incorporation into or synthesis of new tissue occurred during the 2‐week experiment. Our study shows the ability for stable isotopes to provide information about the short‐term or recently incorporated dietary nitrogen and carbon isotopes in crustacean mysids.

Our study provides a foundation for future research into using stable isotope tracers to investigate the ecology of invasive species. Most importantly, applying tracer for isotopic studies does not necessarily change the dietary quality of the food as would be expected when diet‐shift experiments are conducted (Caut et al., 2009). In contrast to traditional dietary shifts, our laboratory‐based dual stable isotope tracer approach (Na^15^NO_3_ and NaH^13^CO_3_) does not shift the experimental organisms into a new diet. This approach is expected to have a minor (if any) dietary effect on isotope values. To our knowledge, no study to date has measured isotopic incorporation in invasive *L. benedeni* using either dietary shift or tracer experiments. In this study, samples of whole‐organism homogenates obtained from fully grown adults in a nonreproductive state were used. Indeed, a longer sampling period would have improved turnover estimates in our study, particularly to give sufficient incorporation time for individuals with slower turnover rates.

Best practices for understanding the ecological patterns of invasive species and their habitats require an understanding of the species’ foraging ecology and habitat. Several field‐based stable isotope studies, particularly those that use isotopic mixing models (Phillips & Gregg, 2003;

Parnell, Inger, Bearhop, & Jackson, 2010) commonly assume (without any a priori information) diet‐tissue steady state. Unfortunately, in cases where the isotopic composition of the animal tissues is not in equilibrium with diet, this can lead to highly misleading conclusion (O'Reilly & Hecky, 2002).

This study offers, for the first time, nitrogen and carbon isotope incorporation rates in the highly invasive mysid species, *L. benedeni* under controlled environmental conditions. Our study provides a foundation for future research into using stable isotope tracers to investigate the ecology of invasive species.

Finally, although more 90% of mysid species are exclusively found in marine habitats, the remaining species represent either species from coastal environment or habitats with direct marine links such as estuaries, coastal rivers, salt‐water caves, or from inland freshwater invasions (Audzijonyte, 2006; Mauchline, 1980). Given invaders move between these environments (which are likely to have differing isotope values), stable isotopes analysis could supply information complementary to high resolution techniques, such as molecular analysis (Gorokhova & Lehtiniemi, 2007), compound‐specific stable isotope (Chikaraishi et al., 2009), and environmental DNA barcoding (Matthew et al., 2016) as indicators of biological invasion. More research into the appropriate scales of the application of natural and tracer‐based isotope approaches within and between systems is highly encouraged.

## Conflict of Interest

None declared.

## References

[ece32928-bib-0201] Adams, T. S. , & Sterner, R. W. (2000). The effect of dietary nitrogen content on trophic level _15_N enrichment. Limnology and Oceanography, 45, 601–607.

[ece32928-bib-0001] Audzijonyte, A. (2006). Diversity and zoogeography of continental mysid crustaceans. Walter and Andre´e de Nottbeck Foundation Scientific Reports, 28, 1–46.

[ece32928-bib-0002] Audzijonyte, A. , Wittmann, K. , Ovcarenko, I. , & Väinölä, R. (2009). The Ponto‐Caspian crustacean *Limnomysis benedeni* dispersing across Europe. Diversity and Distributions, 15, 346–355.

[ece32928-bib-0003] Bacescu, M. (1954). Crustacea. Mysidacea In Fauna Republicii Populare Romine, 4 (pp. 1–126). Bucuresti: Editura Academiei Republicii Populare Romine.

[ece32928-bib-0004] Boecklen, W. J. , Yarnes, C. T. , Cook, B. A. , & James, A. C. (2011). On the use of stable isotopes in trophic ecology. Annual Review of Ecology, Evolution, and Systematics, 42, 411–440.

[ece32928-bib-0005] Borza, P. (2014). Life history of invasive Ponto‐Caspian mysids (Crustacea: Mysida): A comparative study. Limnologica, 44, 9–17.

[ece32928-bib-0007] Caut, S. , Angulo, E. , & Courchamp, F. (2009). Variation in discrimination factors (Δ^15^N and Δ^13^C): The effect of diet isotopic values and applications for diet reconstruction. Journal of Applied Ecology, 46, 443–453.

[ece32928-bib-0008] Chikaraishi, Y. , Ogawa, N. O. , Kashiyama, Y. , Takano, Y. , Suga, H. , Tomitani, A. , … Ohkouchi, N. (2009). Determination of aquatic food‐web structure based on compound‐specific nitrogen isotopic composition of amino acids. Limnology and Oceanography: Methods, 7, 7406–7750.

[ece32928-bib-0009] Fink, P. , & Harrod, C. (2013). Carbon and nitrogen stable isotopes reveal use of pelagic resources by the invasive Ponto‐Caspian Mysid *Limnomysis benedeni* . Isotopes in Environmental and Health Studies, 49, 312–317. 24117428 10.1080/10256016.2013.808197

[ece32928-bib-0010] Fry, B. , & Arnold, C. (1982). Rapid ^13^C/^12^C turnover during growth of brown shrimp (*Penaeus aztecus*). Oecologia, 54, 200–204. 28311429 10.1007/BF00378393

[ece32928-bib-0011] Gannes, L. Z. , O'Brien, D. M. , & Martínez del Rio, C. (1997). Stable isotopes in animal ecology: Assumptions, caveats and a call for more laboratory experiments. Ecology, 78, 1271–1276.

[ece32928-bib-0012] Gergs, R. , Hanselmann, A. J. , Eisele, I. , & Rothhaupt, K.‐O. (2008). Autecology of *Limnomysis benedeni* Czerniavsky, 1882 (Crustacea: Mysida) in Lake Constance, Southwestern Germany. Limnologica, 38, 139–146.

[ece32928-bib-0013] Gorokhova, E. , & Lehtiniemi, M. (2007). A combined approach to understand trophic interactions between Cercopagis pengoi (Cladocera: Onychopoda) and mysids in the Gulf of Finland. Limnology and Oceanography, 52, 685–695.

[ece32928-bib-0203] Guillard, R. (1975). Culture of phytoplankton for feeding marine invertebrates In Smith W., & Chanley M. (Eds.), Culture of marine invertebrate animals (pp. 29–60). New York: Plenum Press.

[ece32928-bib-0014] Hänfling, B. , Edwards, F. , & Gherardi, F. (2011). Invasive alien Crustacea: Dispersal, establishment, impact and control. Biological Control, 56, 573–595.

[ece32928-bib-0015] Hanselmann, A. J. , Gergs, R. , & Rothhaupt, K.‐O. (2011a). Embryonic development time of the freshwater mysid *Limnomysis benedeni* Czerniavsky as a function of water temperature. Aquatic Ecology, 45, 539–546.

[ece32928-bib-0016] Hanselmann, A. J. , Gergs, R. , & Rothhaupt, K.‐O. (2011b). Seasonal shifts in the life cycle of the ponto‐caspian invader *Limnomysis benedeni* (Crustacea: Mysida): A physiological adaptation? Hydrobiologia, 673, 193–204.

[ece32928-bib-0017] Hanselmann, A. J. , Hodapp, B. , & Rothhaupt, K.‐O. (2013). Nutritional ecology of the invasive freshwater mysid *Limnomysis benedeni*: Field data and laboratory experiments on food choice and juvenile growth. Hydrobiologia, 705, 75–86.

[ece32928-bib-0018] Hesslein, R. H. , Hallard, K. A. , & Ramlal, P. (1993). Replacement of sulfur, carbon, and nitrogen in tissue of growing broad whitefish (*Coregonus nasus*) in response to a change in diet traced by ^34^S, ^13^C, and ^15^N. Canadian Journal of Fisheries and Aquatic Science, 50, 2071–2076.

[ece32928-bib-0204] Jackson, M. C. , Grey, J. , Miller, K. , Britton, J. R. , & Donohue, I. (2016). Dietary niche constriction when invaders meet natives: evidence from freshwater decapods. Journal of Animal Ecololgy, 85, 1098–1107. 10.1111/1365-2656.12533 27084460

[ece32928-bib-0021] Kaufman, M. R. , Gradinger, R. R. , Bluhm, B. A. , & O'Brien, D. M. (2008). Using stable isotopes to assess carbon and nitrogen turnover in the Arctic sympatric amphipod *Onisimus litoralis* . Oecologia, 158, 11–22. 18709389 10.1007/s00442-008-1122-y

[ece32928-bib-0022] Marín Leal, J. C. , Dubois, S. , Orvain, F. , Galois, R. , Blin, J.‐L. , Ropert, M. , … Lefebvre, S. (2008). Stable isotopes (δ^13^C, δ^15^N) and modelling as tools to estimate the trophic ecology of cultivated oysters in two contrasting environments. Marine Biology, 153, 673–688.

[ece32928-bib-0023] Martínez del Rio, C. , & Anderson‐Sprecher, R. (2008). Beyond the reaction progress variable: The meaning and significance of isotopic incorporation data. Oecologia, 156, 765–772. 18446374 10.1007/s00442-008-1040-z

[ece32928-bib-0024] Martínez del Rio, C. , Wolf, N. , Carleton, S. A. , & Gannes, L. Z. (2009). Isotopic ecology ten years after a call for more laboratory experiments. Biological reviews of the Cambridge Philosophical Society, 84, 91–111. 19046398 10.1111/j.1469-185X.2008.00064.x

[ece32928-bib-0025] Matthew, M. D. , Larson, E. R. , Renshaw, M. A. , Gantz, C. A. , Egan, S. P. , Erickson, D. M. , & Lodge, D. M. (2016). Environmental DNA (eDNA) detects the invasive rusty crayfish *Orconectes rusticusat* low abundances. Journal of Applied Ecology, 53, 722–732. 27773942 10.1111/1365-2664.12621 PMC5053277

[ece32928-bib-0026] Mauchline, J. (1980). The biology of mysids and euphausiids. Advances in Marine Biology, 18, 1–680.

[ece32928-bib-0027] O'Reilly, C. M. , & Hecky, R. E. (2002). Interpreting stable isotopes in food webs: Recognizing the role of time averaging at different trophic levels. Limnology and Oceanography, 47, 306–309.

[ece32928-bib-0028] Parnell, A. C. , Inger, R. , Bearhop, S. , & Jackson, A. L. (2010). Source partitioning using stable isotopes: coping with too much variation. PLoS ONE, 5(3), e9672. 20300637 10.1371/journal.pone.0009672 PMC2837382

[ece32928-bib-0029] Phillips, D. L. , & Gregg, J. W. (2003). Source partitioning using stable isotopes: Coping with too many sources. Oecologia, 136, 261–269. 12759813 10.1007/s00442-003-1218-3

[ece32928-bib-0206] Post, D. M. (2002). Using stable isotopes to estimate trophic position: models, methods, and assumptions. Ecology, 83, 703–718.

[ece32928-bib-0031] Rothhaupt, K.‐O. , Hanselmann, A. J. , & Yohannes, E. (2014). Niche differentiation between sympatric alien aquatic crustaceans: An isotopic evidence. Basic and Applied Ecology, 15, 453–463.

[ece32928-bib-0032] Suring, E. , & Wing, S. R. (2009). Isotopic turnover rate and fractionation in multiple tissues of red rock lobster (*Jasus edwardsii*) and blue cod (*Parapercis colias*): Consequences for ecological studies. Journal of Experimental Marine Biology and Ecology, 370, 56–63.

[ece32928-bib-0033] Tieszen, L. L. , Boutton, T. W. , Tesdahl, K. G. , & Slade, N. A. (1983). Fractionation and turnover of stable carbon isotopes in animal‐tissues—implications for delta‐C‐13 analysis of diet. Oecologia, 57, 32–37. 28310153 10.1007/BF00379558

[ece32928-bib-0034] Wittmann, K. J. , & Ariani, A. P. (2009). Reappraisal and range extension of non‐indigenous Mysidae (Crustacea, Mysida) in continental and coastal waters of eastern France. Biological Invasions, 11, 401–407.

